# iPiDA-LTR: Identifying piwi-interacting RNA-disease associations based on Learning to Rank

**DOI:** 10.1371/journal.pcbi.1010404

**Published:** 2022-08-15

**Authors:** Wenxiang Zhang, Jialu Hou, Bin Liu

**Affiliations:** 1 School of Computer Science and Technology, Beijing Institute of Technology, Beijing, China; 2 Advanced Research Institute of Multidisciplinary Science, Beijing Institute of Technology, Beijing, China; University of Electronic Science and Technology, CHINA

## Abstract

Piwi-interacting RNAs (piRNAs) are regarded as drug targets and biomarkers for the diagnosis and therapy of diseases. However, biological experiments cost substantial time and resources, and the existing computational methods only focus on identifying missing associations between known piRNAs and diseases. With the fast development of biological experiments, more and more piRNAs are detected. Therefore, the identification of piRNA-disease associations of newly detected piRNAs has significant theoretical value and practical significance on pathogenesis of diseases. In this study, the iPiDA-LTR predictor is proposed to identify associations between piRNAs and diseases based on Learning to Rank. The iPiDA-LTR predictor not only identifies the missing associations between known piRNAs and diseases, but also detects diseases associated with newly detected piRNAs. Experimental results demonstrate that iPiDA-LTR effectively predicts piRNA-disease associations outperforming the other related methods.

## Author summary

Accumulating evidences have indicated that dysfunction and abnormal expression of piRNAs are closely associated with the emergence and development of diseases. Currently, identifying piRNA-disease associations mainly focuses on biological experimental methods and computational methods. However, biological experimental methods take substantial time and resources. Computational methods mainly focused on identifying diseases associated known piRNAs. With the development of biological technology, more and more newly detected piRNAs were detected. Therefore, identifying diseases associated with newly detected piRNAs is more important compared with identifying diseases associated with known piRNAs. Information retrieval (IR)’s goal is to rank documents based on the relevance to certain topics. This task is particularly similar with identification of piRNA-disease associations. Specifically, ranking documents related to previous topics corresponds to identify diseases associated with known piRNAs, and ranking documents related to novel topics is similar to identify diseases associated with newly detected piRNAs. Therefore, we propose a new predictor called iPiDA-LTR to predict associations between piRNAs and diseases based on information retrieval technology. Experimental results indicated that iPiDA-LTR is promising in identifying diseases associated with known piRNAs and newly detected piRNAs.

This is a *PLOS Computational Biology* Methods paper.

## Introduction

Piwi-interacting RNA (piRNA) with 24–31 nucleotides in length is a class of small RNAs interacting with Piwi-subfamily Argonaute proteins [1–3]. Early studies found that piRNAs mainly located in germ stem cells on drosophila and mouse, and regulated germ stem cell proliferation [4–6]. With the fast development of biotechnology and computing techniques [7, 8], more and more piRNAs were discovered, and the corresponding functions were also detected, including stem cell proliferation, gene expression, and heterochromatin formation, etc [9–12].

As more and more piRNA functions were detected, many evidences indicated that dysfunction and abnormal expression of piRNAs are closely associated with the emergence and development of diseases [13–17]. Therefore, the identification of associations between piRNAs and diseases is important for diagnosis and treatment of diseases [18, 19]. Currently, it mainly focused on biological experimental methods and computational methods. For biological experiments methods, Cabral et al. indicated that piRNAs play a role in the process of translational research of gastric cancer as potential biomarkers [20]. Krishnan et al. identified eight non-redundant piRNAs as breast cancer markers [21]. Roy et al. studied the reciprocal expression between piRNAs and the corresponding targets, and provided a novel insight into the role of piRNAs in Alzheimer’s disease [22]. Although biological experimental methods are highly reliable, it takes substantial time and resources. Some computational methods have been proposed for identifying the associations between non-coding RNAs and diseases, such as miRNA-disease associations [23], circRNA-disease associations [24], etc. In this regard, computational methods are proposed to predict piRNA-disease associations, which can serve as powerful auxiliary tools to save time and cost compared with biological experiments. For example, Wei et al. proposed the first computational predictor for identifying piRNA-disease associations based on the positive unlabelled learning algorithm, and established the first web server [25]. A convolutional neural network was utilized to extract association features between piRNAs and diseases, and then the Support Vector Machine was employed to construct the predictor [26]. Although computational methods have been proposed, they mainly aim at the application scenario of identifying missing associations between known piRNAs and diseases. However, more and more newly detected piRNAs were detected [27–29]. Therefore, the application scenario of identifying piRNA-disease associations of newly detected piRNAs is very important to investigate piRNA functions and disease pathogenesis.

**Fig 1:**
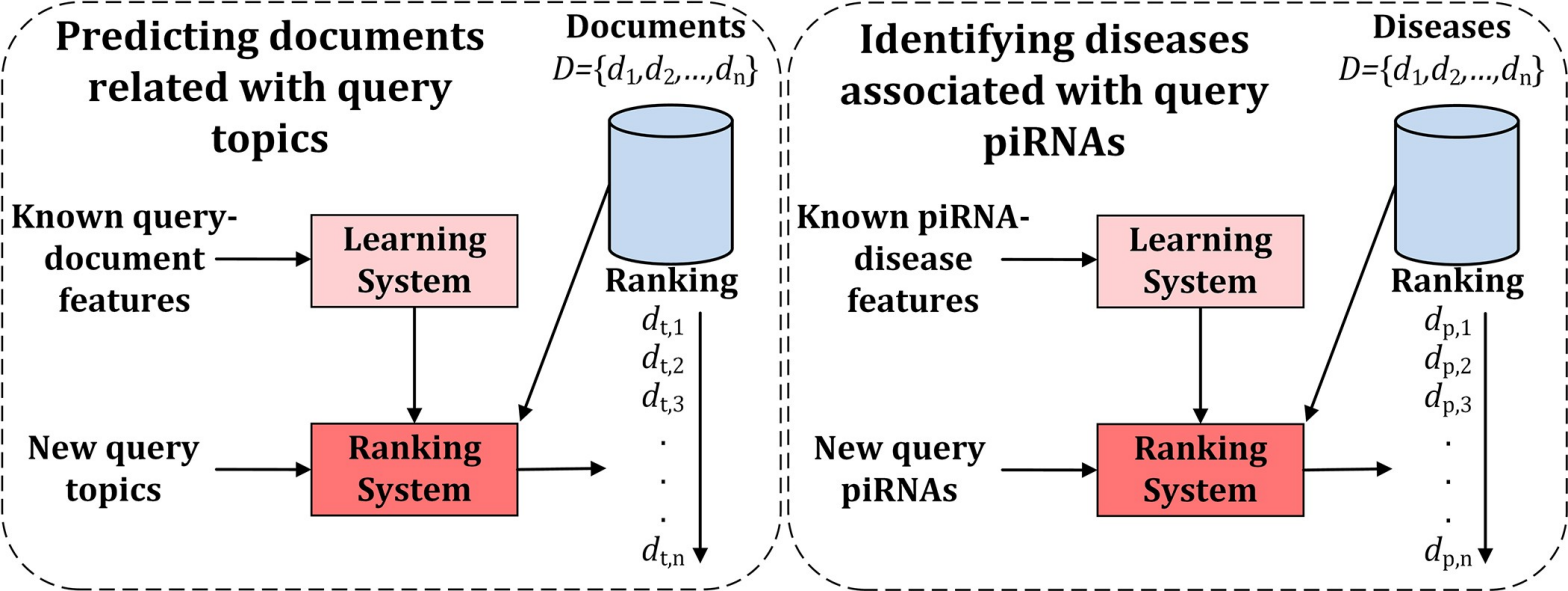
The similarities between the prediction of documents related with query topics and the identification of diseases associated with query piRNAs, where piRNA and disease can be treated as query and document, respectively.

In recent years, information retrieval (IR) becomes a widely used technology, whose ultimate goal is to rank documents based on the relevance to certain topics [30, 31]. As an successful algorithm in information retrieval, Learning to Rank (LTR) [32, 33] has been successfully applied to web page retrieval employed by Google [34], Yahoo [35], Microsoft [36], etc. Compared with traditional IR methods, the advantage of LTR is that it integrates component methods so as to automatically rank documents associated with query from multiple perspectives [30]. LTR has been applied in identifying circRNA-disease associations [24], detecting protein remote homology [37], predicting protein-phenotype associations [38], drug–target binding affinity prediction [39], etc. The core concept of LTR is to calculate the relevance score *f*(*q*, *d*) between query *q* and document *d*. Therefore, this task is particularly similar with identification of piRNA-disease associations (see **Fig 1**). PiRNAs and diseases can be treated as queries and documents, respectively. Learning to Rank not only identifies associations between known piRNAs and diseases, but also ranks diseases associated with newly detected piRNAs.

In this study, we propose a new predictor, named iPiDA-LTR, to predict associations between piRNAs and diseases, which has the following advantages. iPiDA-LTR predictor combines component methods and Learning to Rank, which cannot only identify missing associations between known piRNAs and diseases, but also can identify diseases associated with newly detected piRNAs. Experimental results indicated that iPiDA-LTR is promising to identify piRNA-disease associations. A web server of iPiDA-LTR is constructed to identify diseases associated with query piRNAs, which can be accessed at http://bliulab.net/iPiDA-LTR.

## Materials and methods

### Materials

To imitate two application scenarios, we construct two types of datasets based on piRDisease v1.0 database [40] collecting 7939 piRNA-disease associations with 4796 piRNAs and 28 diseases. Firstly, a standard dataset $S_{all}$ is constructed following [25], which can be represented as:

$$\left\{ \begin{array}{c} S_{all}=A_{all}\cup P_{all}\cup D\\ A_{all}=A_{all}^{+}\cup A_{all}^{-} \end{array}\right.$$ (1)

where $A_{all}$ represents 5002 piRNA-disease associations from [25]. $P_{all}$ and D contain 4350 piRNAs and 21 diseases from $A_{all}$, respectively. $A_{all}^{+}$ and $A_{all}^{-}$ contain known piRNA-disease associations and unknown piRNA-disease associations, respectively. Specifically, piRNA-disease associations contained in $A_{all}^{+}$ are labelled as 1, otherwise 0. To avoid overfitting problem, $S_{all}$ is further divided into a benchmark dataset and an independent dataset. The benchmark dataset is used to adjust parameters and train model via cross-validation, and the independent dataset is employed to evaluate the performance of different methods.

### For the first application scenario: predicting associations between known piRNAs and known diseases

Benchmark dataset and independent dataset are constructed as:

$$\left\{ \begin{array}{c} S_{ben}^{a}=S_{ben}^{a+}\cup S_{ben}^{a-}\\ S_{ind}^{a}=S_{ind}^{a+}\cup S_{ind}^{a-}\\ S_{all}^{+}=S_{ben}^{a+}\cup S_{ind}^{a+}\\ S_{all}^{-}=S_{ben}^{a-}\cup S_{ind}^{a-} \end{array}\right.$$ (2)

where we randomly select 20% associations from $A_{all}^{+}$ and $A_{all}^{-}$ to construct $S_{ind}^{a+}$ and $S_{ind}^{a-}$, respectively, and then the remaining associations in $A_{all}^{+}$ and $A_{all}^{-}$ are used to construct $S_{ben}^{a+}$ and $S_{ben}^{a-}$, respectively. Obviously, $S_{ben}^{a}$ represents benchmark dataset, which is used to optimize parameters and train models, and then trained models are used to identify unknown associations in $S_{ind}^{a}$.

### For the second application scenario: predicting the associations between newly detected piRNAs and known diseases

To imitate the second application scenario, we randomly select 80% and 20% piRNAs from $P_{all}$ as known piRNA set $P_{all}^{knwon}$ and newly detected piRNA set $P_{all}^{unknown}$, respectively, based on which benchmark dataset and independent dataset are constructed as:

$$\left\{ \begin{array}{c} S_{ben}^{p}=\{S_{ben}^{p+}\cup S_{ben}^{p-}|piRNAs\in P_{all}^{knwon}\}\\ S_{ind}^{p}=\{S_{ind}^{p+}\cup S_{ind}^{p-}|piRNAs\in P_{all}^{unknown}\}\\ S_{all}^{+}=S_{ben}^{p+}\cup S_{ind}^{p+}\\ S_{all}^{-}=S_{ben}^{p-}\cup S_{ind}^{p-} \end{array}\right.$$ (3)

where $S_{ben}^{p}$ and $S_{ind}^{p}$ represent benchmark dataset and independent dataset, respectively. PiRNAs contained in $S_{ben}^{p}$ and $S_{ind}^{p}$ belong to $P_{all}^{known}$ and $P_{all}^{unknown}$, respectively. Detailed information of $S_{ben}^{a}$, $S_{ind}^{a}$, $S_{ben}^{p}$ and $S_{ind}^{p}$ is shown in **Table 1**. The datasets can be obtained at http://bliulab.net/iPiDA-LTR/dataset/.

**Table 1:** The detailed statistical information of $S_{ben}^{a}$, $S_{ind}^{a}$, $S_{ben}^{p}$ and $S_{ind}^{p}$.

Datasets	PiRNAs	Diseases	Known*	Unknown^#^
$S_{ben}^{a}$	4350	21	4002	69079
$S_{ind}^{a}$	4311	21	1000	17269
$S_{ben}^{p}$	3480	21	3999	69081
$S_{ind}^{p}$	870	21	1003	17267

^*^ The associations between piRNAs and diseases have been validated by experiments

^**#**^ The associations between piRNAs and diseases without experiment validations.

### Method overview

**Fig 2:**
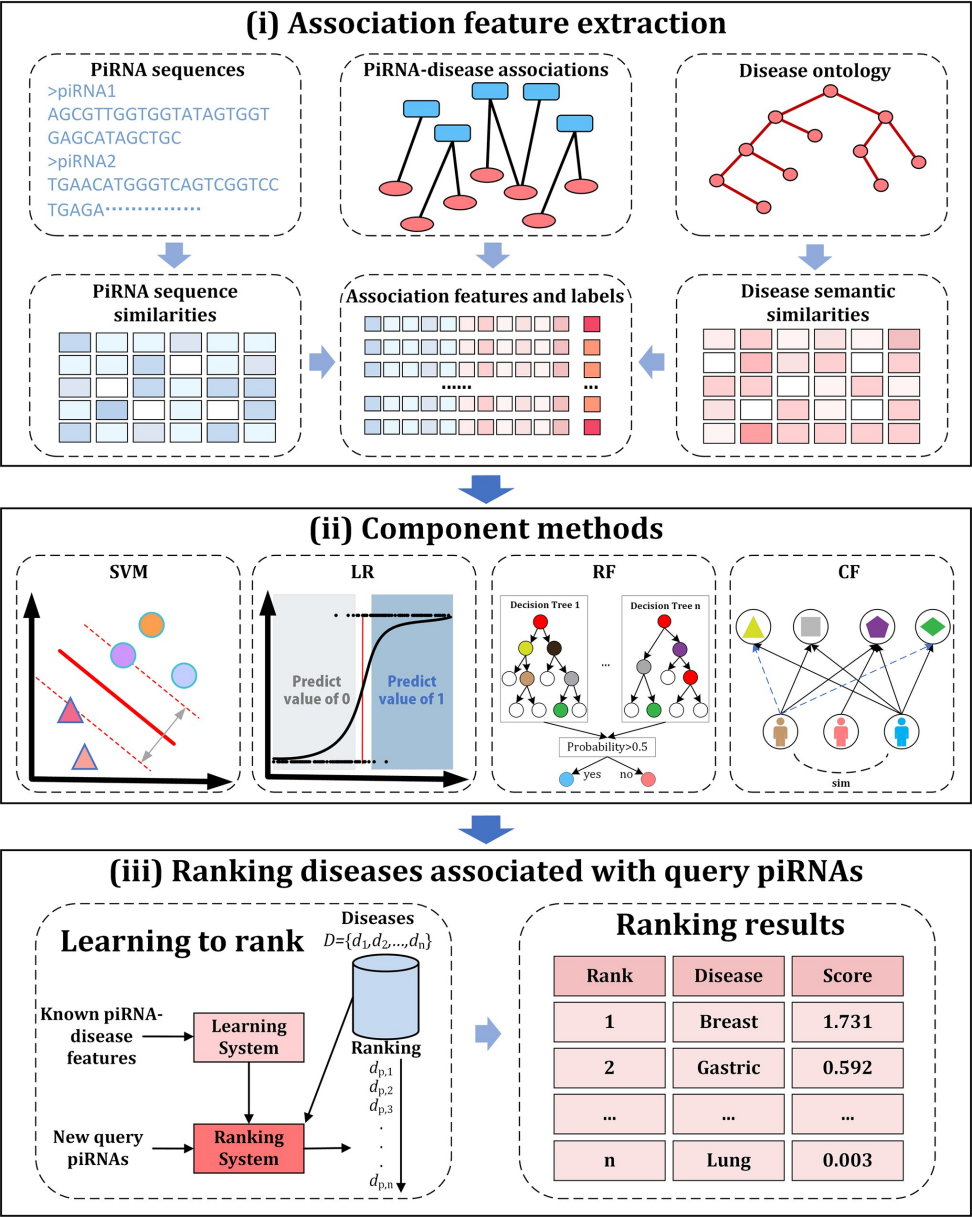
The workflow of iPiDA-LTR predictor. (i) Association feature extraction: piRNA sequences and disease ontology are used to calculate piRNA sequence similarities and disease semantic similarities by combining them and piRNA-disease associations to construct association features and labels; (ii) Component methods: four methods are used to train models with benchmark dataset, and then trained models are utilized to calculate association scores of query piRNAs; (iii) Ranking diseases associated with query piRNAs: association scores of samples in the benchmark dataset are used to train LambdaMART model, and then trained LambdaMART model is employed to rank diseases associated with query piRNAs.

In this study, a novel ranking framework, named iPiDA-LTR, is proposed to solve two application scenarios. The workflow of iPiDA-LTR is shown in **Fig 2** with three steps: (a) Association feature extraction; (b) Component methods; (c) Ranking diseases associated with query piRNAs.

### Association feature extraction

#### PiRNA sequence similarities

The piRNA similarities play a vital role in RNA-disease association identification [24–26], and piRNA sequence similarities have been applied to piRNA-disease association identification [25, 26]. Many methods have been proposed to calculate sequence similarities [41–43]. For example, Smith-Waterman algorithm has been successfully applied to multiple sequence analysis tasks, including RNA sequence similarity analysis [25, 26, 44], protein sequence analysis [45, 46], etc. In this study, we employ Smith-Waterman algorithm [41, 44] to calculate piRNA sequence similarities:

$$S_{P}(p_{i},p_{j})=\frac{SW(p_{i},p_{j})}{\sqrt{SW(p_{i},p_{i})\times SW(p_{j},p_{j})}}$$ (4)

where **S**_**P**_(*p*_*i*_, *p*_*j*_) is similarity between piRNA *p*_*i*_ and piRNA *p*_*j*_. SW(*p*_*i*_, *p*_*j*_) represents local alignment score between piRNA *p*_*i*_ and piRNA *p*_*j*_ based on Smith-Waterman algorithm.

#### Disease semantic similarities

The disease semantic similarity calculation is a key component in RNA-disease association identification. The disease ontology [47] has been applied to RNA-disease association identification so as to calculate disease semantic similarities [48–53]. Disease ontology organized by the directed acyclic graph (DAG) provides a hierarchical structure of the complex disease parent node [47]. Similar diseases share similar hierarchical structure in DAG of disease ontology. Therefore, DAG of disease ontology helps to measure similarity between two diseases. In this study, we use DAG of disease ontology to calculate disease semantic similarities [54, 55]:

$$S_{D}(m,n)=\frac{\sum_{i\in T_{m}\cap T_{n}}\left(S_{m}(i)+S_{n}(i)\right)}{\sum_{j\in T_{m}}S_{m}(j)+\sum_{j\in T_{n}}S_{n}(j)}$$ (5)

$$\left\{ \begin{array}{cc} S_{n}(i)=max\{0.5*S_{n}(j)|j\in children\;of\;i\} & if\;i\neq n\\ S_{n}(i)=1 & if\;i=n \end{array}\right.$$ (6)

where **S**_**D**_(*m*, *n*) is similarity between disease *m* and disease *n*. T_*k*_ represents the node set containing the ancestor nodes of *k* and itself. S_*n*_(*i*) is the semantic value of node *i* to node *n*.

#### Association features and labels

The association feature between disease *d* and the query piRNA *p* is:

$$F(p,d)=\{S_{P}(p,:),S_{D}(d,:)\}$$ (7)

where F(p,d) is the association features of piRNA *p* and disease *d*. **S**_**P**_(*p*,:) and **S**_**D**_(*d*,:) represent *p*th row and *d*th row in the **S**_**P**_ and **S**_**D**_, respectively. If piRNA *p* is associated with disease *d*, the label of F(p,d) is equal to 1, otherwise 0.

### Component methods

In this study, we select two types of component methods to calculate association scores, including machine learning methods and collaborative filtering (CF). For machine learning methods, Random Forest (RF) [56–60], Logistic Regression method (LR) [61], and Support Vector Machine (SVM) [62–64] are employed, treating the identification of piRNA-disease association as a classification problem. CF is a recommendation algorithm [65, 66], which utilizes guilt-by-association assumption to identify piRNA-disease association focusing on local information. In this study, association features of benchmark dataset (see **Eq 7**) are used to train machine learning models, and then used to calculate association scores for $S_{all}$ dataset. Finally, association features between piRNA *p* and disease *d* can be represented as:

$$Q(p,d)=\{V_{CF}(p,d),V_{LR}(p,d),V_{RF}(p,d),V_{SVM}(p,d)\}$$ (8)

where Q(p,d) represents association features of piRNA *p* and disease *d*. $V_{CF}(p,d)$, $V_{LR}(p,d)$, $V_{RF}(p,d)$ and $V_{SVM}(p,d)$ are association scores between piRNA *p* and disease *d* calculated by CF, LR, RF and SVM, respectively.

### Ranking diseases associated with query piRNAs

In this study, we employ Learning to Rank (LTR) to solve the problem of identifying potential piRNA-disease associations motivated by information retrieval [24, 37, 38, 67]. LTR is generally classified into three categories, including ListWise, PairWise and PointWise [68]. In this study, a ListWise method LambdaMART [32] is selected to obtain high quality of top-ranked diseases, which has been applied in identifying circRNA-disease associations [24], detecting protein remote homology [37], predicting protein-phenotype associations [38] and drug–target binding affinity prediction [39]. The number of trees, the truncation level *k*, shrinkage and the number of leaves are the four main parameters. The truncation level of *k* influences the quality of top-ranked results by Normalized Discounted Cumulative Gain (NDCG), which can be formulated as [32]:

$$\left\{ \begin{array}{c} DCG@k=\sum_{i=1}^{k}\frac{2^{{rel}_{i}}-1}{{log}_{2}(i+1)}\\ NDCG@k=\frac{DCG@k}{IDCG@k} \end{array}\right.$$ (9)

where *k* represents the truncation level. IDCG@*k* is the value of DCG@*k* in the best optimal ranking results. If a query piRNA is associated with disease located in position *i*, *rel*_*i*_ is equal to 1, otherwise 0. To obtain the final ranking results, association scores calculated by **Eq 8** for training set are used to train LambdaMART model, and the trained LambdaMART model is employed to rank diseases associated with query piRNAs based on association scores of query piRNAs.

## Results and discussion

### Evaluation criteria

In this study, the benchmark dataset is employed to optimize the parameters of the models, and the independent dataset is used to evaluate the performance of predictors. How to evaluate the ranking quality and prediction performance is crucial for identifying piRNA-disease associations. Because iPiDA-LTR predictor treats the identification of piRNA-disease associations as an information retrieval ranking task, we employ three important ranking criteria to evaluate the rank quality of different predictors: Normalized Discounted Cumulative Gain (NDCG), Mean Average Precision (MAP) and ROC*k*. Besides, Area Under the ROC Curve (AUC) and Area Under the Precision-Recall Curve (AUPR) are also used to measure comprehensive performance [69–72]. The average values of these criteria for all query piRNAs are calculated to evaluate performance of predictors.

### The effect of parameters for identifying piRNA-disease associations

**Fig 3:**
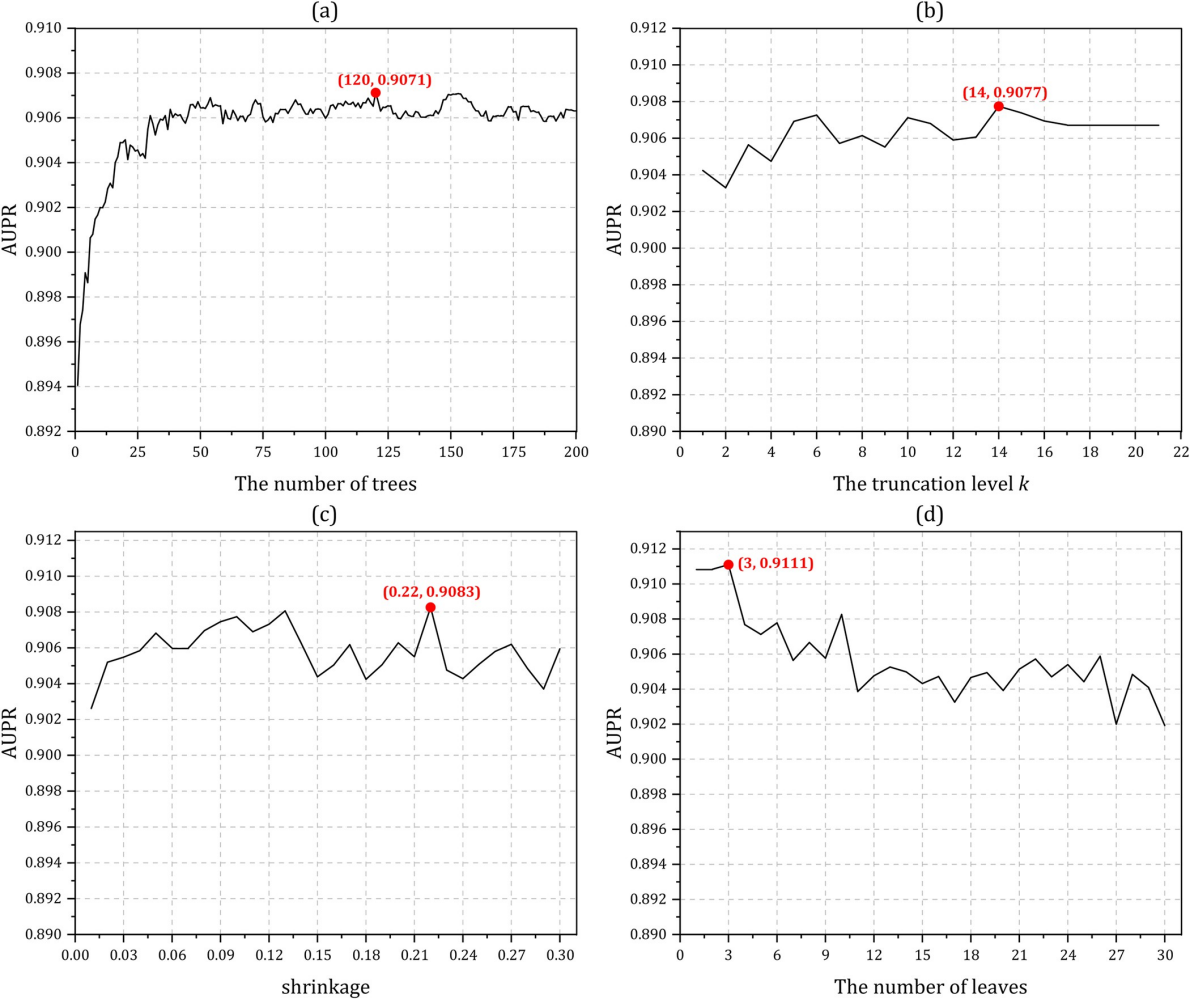
The predictive results of iPiDA-LTR predictor on $S_{ben}^{a}$ dataset via five-fold cross-validation. (a) The truncation level *k*, shrinkage and the number of leaves are assigned as 10, 0.10, and 10 respectively, which are RankLib’s default values (https://sourceforge.net/p/lemur/wiki/RankLib/), and the optimal value of the number of trees is 120; (b) The number of trees, shrinkage and the number of leaves are fixed as 120, 0.10 and 10 respectively, and the truncation level *k* is optimized as 14; (c) The number of trees, the truncation level *k* and the number of leaves are fixed as 120, 14 and 10 respectively, and the shrinkage is optimized as 0.22; (d) The number of trees, the truncation level *k* and shrinkage are 120, 14 and 0.22 respectively, and the number of leaves is set as 3.

**Fig 4:**
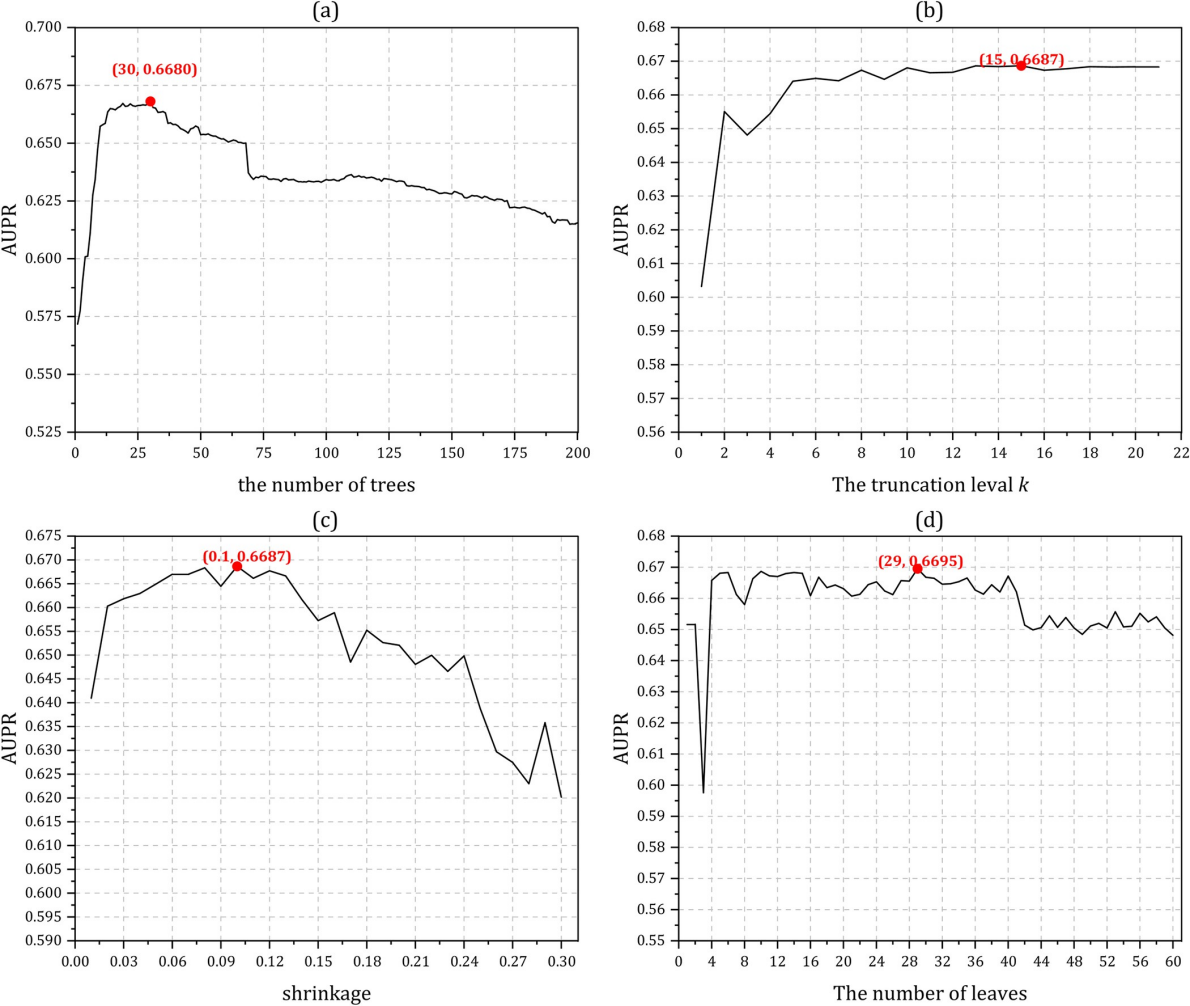
The predictive results of iPiDA-LTR predictor on $S_{ben}^{p}$ dataset via five-fold cross-validation. (a) The truncation level *k*, shrinkage and the number of leaves are assigned as 10, 0.10, and 10 respectively, which are the RankLib’s default values (https://sourceforge.net/p/lemur/wiki/RankLib/), and the optimal value of the number of trees is 30; (b) The number of trees, shrinkage and the number of leaves are fixed as 30, 0.10 and 10 respectively, and the truncation level *k* is optimized as 15; (c) The number of trees, the truncation level *k* and the number of leaves are fixed as 30, 15 and 10 respectively, and the shrinkage is optimized as 0.10; (d) The number of trees, the truncation level *k* and shrinkage are 30, 15 and 0.1 respectively, and the number of leaves is optimized as 29.

iPiDA-LTR predictor mainly contains the following four parameters: the number of trees, the truncation level *k*, shrinkage and the number of leaves. Due to the large number of combinations of the four parameters, we fix three parameters in turns, and then find the local optimal values of the remaining parameters according to AUPR. The influences of different combinations of parameters for iPiDA-LTR on $S_{ben}^{a}$ dataset and $S_{ben}^{p}$ dataset are shown in **Figs 3** and **4**, respectively, from which we can see that the final optimized combinations of four parameters on iPiDA-LTR predictor on $S_{ben}^{a}$ dataset and $S_{ben}^{p}$ dataset are (120, 14, 0.22, 3) and (30, 15, 0.10, 29), respectively.

### Complementary analysis for component methods

In this study, iPiDA-LTR incorporates two types of component methods, including machine learning methods (LR, RF and SVM) and collaborative filtering (CF). LR, RF and SVM are obtained by python package Scikit-learn [73]. For LR’s parameters, max_iter and solver are assigned as 300 and liblinear, respectively. For RF’s parameters, n_estimators, max_leaf_nodes, n_jobs and max_features are assigned as 80, 10, -1 and 0.2, respectively. For SVM’s parameters, kernel and probability are assigned as linear and True, respectively. We analyze the impact of different types of component methods to identify associations between piRNAs and diseases, and the results are shown in **Tables 2** and **3**, from which we can see the followings: (i) iPiDA-LTR predictor outperforms iPiDA-LTR-ML predictor on $S_{ben}^{a}$ dataset and $S_{ben}^{p}$ dataset; (ii) The iPiDA-LTR obviously outperforms iPiDA-LTR-ML in terms of ranking criteria (NDCG@5 and ROC1), especially for the second application scenario (see **Table 3**). Machine learning methods based on classification algorithms focus on global predictive performance, and collaborative filtering can identify special piRNA-related diseases focusing on local predictive performance. Therefore, machine learning methods and collaborative filtering are complementary. It is not surprising that iPiDA-LTR predictor obtains the best performance compared with iPiDA-LTR-ML, because iPiDA-LTR shares the advantages of these two types of methods.

**Table 2:** The comparison results of predictors based on Learning to Rank integrating different component methods via five-fold cross-validation on $S_{ben}^{a}$ dataset.

	AUC	AUPR	NDCG@5	MAP	ROC1	ROC3	ROC5
**iPiDA-LTR-ML** ^**a**^	0.9511	0.9003	0.9492	0.9305	0.8678	0.9407	0.9503
**iPiDA-LTR** ^**b**^	0.9543	0.9111	0.9545	0.9379	0.8822	0.9457	0.9538

^a^ The component methods include RF, LR and SVM

^b^ The component methods include RF, LR, SVM and CF.

**Table 3:** The comparison results of predictors based on Learning to Rank integrating different component methods via five-fold cross-validation on and $S_{ben}^{p}$ dataset.

	AUC	AUPR	NDCG@5	MAP	ROC1	ROC3	ROC5
**iPiDA-LTR-ML** ^**a**^	0.9544	0.6243	0.7722	0.7299	0.5103	0.7779	0.8478
**iPiDA-LTR** ^**b**^	0.9558	0.6695	0.7884	0.7581	0.5725	0.7918	0.8515

^a^ The component methods include RF, LR and SVM

^b^ The component methods include RF, LR, SVM and CF.

**Fig 5:**
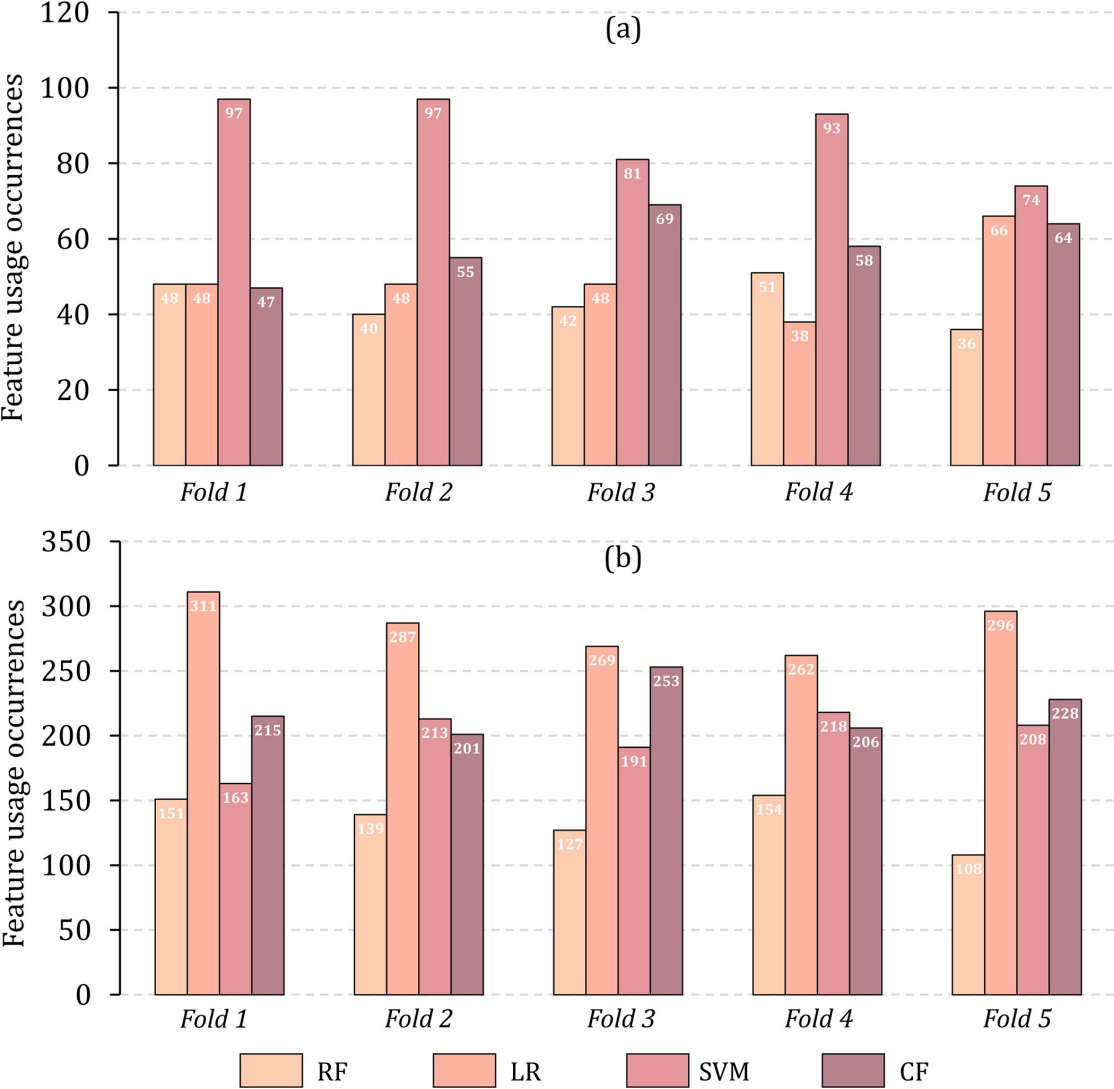
The usage frequencies of component methods, calculated by Apache commons-math3 library and RankLib library. (a) shows the usage frequencies of component methods on $S_{ben}^{a}$ dataset via five-fold cross-validation; (b) shows the usage frequencies of component methods on $S_{ben}^{p}$ dataset via five cross-validation.

The usage frequencies of component methods measure the contribution of component methods for iPiDA-LTR. **Fig 5** shows the usage frequencies of component methods on iPiDA-LTR, from which we can see that each component method is frequently used, indicating that they are important for iPiDA-LTR. **Tables 2** and **3** and **Fig 5** show that component methods are complementary, and iPiDA-LTR combines them leading to better performance for identifying piRNA-disease associations.

### Comparison with related methods

**Fig 6:**
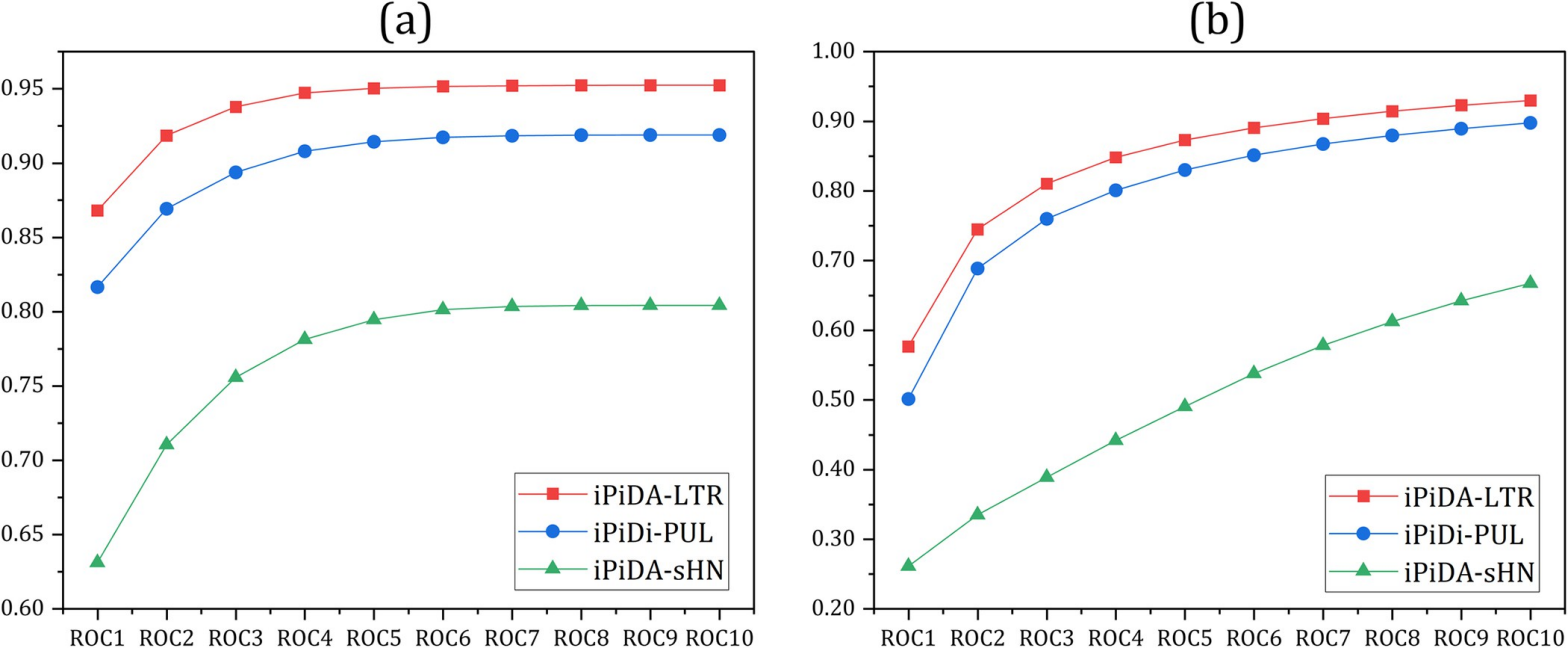
The comparison results of different methods. (a) and (b) are based on $S_{ind}^{a}$ dataset and $S_{ind}^{p}$ dataset, respectively.

In this section, the two state-of-the-art predictors including iPiDi-PUL predictor [25] and iPiDA-sHN predictor [26] are compared with iPiDA-LTR predictor, and the results are shown in **Tables 4** and **5**, from which we can see that iPiDA-LTR is better than the other methods, indicating that iPiDA-LTR is more suitable for identifying piRNA-disease associations. Researchers tend to focus on the top ranked predicted associations in practical application scenarios. Therefore, we analyze the quality of the predicted results (see **Fig 6**), from which we can see that iPiDA-LTR outperforms the other predictors in terms of ROC1-ROC10. It is not surprising because the loss function of LambdaMART NDCG mainly focuses on the top-ranked predictive known associations (see **Eq 9**).

**Table 4:** The comparison results between iPiDA-LTR and two state-of-the-art predictors on $S_{ind}^{a}$ dataset.

	AUC	AUPR	NDCG@5	MAP
**iPiDi-PUL**	0.9153	0.8511	0.9190	0.8847
**iPiDA-sHN**	0.8042	0.7023	0.8198	0.7705
**iPiDA-LTR**	0.9521	0.8987	0.9472	0.9283

Note: iPiDi-PUL and iPiDA-sHN are reproduced, and their parameters are set as the optimized values reported in [25] and [26], respectively.

**Table 5:** The comparison results between iPiDA-LTR and two state-of-the-art $S_{ind}^{p}$ dataset.

	AUC	AUPR	NDCG@5	MAP
**iPiDi-PUL**	0.9413	0.6154	0.7736	0.7110
**iPiDA-sHN**	0.8015	0.3702	0.4875	0.4583
**iPiDA-LTR**	0.9623	0.6780	0.8067	0.7697

Note: iPiDi-PUL and iPiDA-sHN are reproduced, and their parameters are set as the optimized values reported in [25] and [26], respectively.

### Case study

To illustrate the predictive performance of iPiDA-LTR predictor for the identification of associations between new piRNAs and diseases, two query piRNAs, including piR-hsa-23210 and piR-hsa-15023, are selected as query piRNAs from $S_{all}$ dataset, respectively. The remaining piRNAs in $S_{all}$ are used to train iPiDA-LTR model, and then the trained iPiDA-LTR model is employed to predict diseases associated with piR-hsa-15023 and piR-hsa-23210.

The predicted results of piR-hsa-23210 and piR-hsa-15023 are shown in **Tables 6** and **7,** respectively, from which we can see the followings: (i) The evidences for the top five predicted piR-hsa-23210-associated diseases are supported by PubMed (https://pubmed.ncbi.nlm.nih.gov/). For example, the target gene of piR-hsa-23210 is SMC5, which plays crucial roles in the process of human spermatogenesis, such as on the synaptonemal complex between synapsed chromosomes, and in the development of spermatogonial cells [74]. Roy et al. found that piR-33044 (piR-hsa-23210) is significantly abnormal expression in Alzheimer Disease [22]. (ii) Four diseases in **Table 7** have been proved to be associated with piR-hsa-15023. For example, Busch et al. found that piR-hsa-15023 is down-regulated in renal cell carcinoma [75]. piR-hsa-15023 showed a significantly differentially expression in gastric adenocarcinoma and non-malignant stomach tissue [76]. Therefore, these results demonstrated that iPiDA-LTR predictor is an effective approach to identify associated diseases for newly detected query piRNAs.

**Table 6:** The top five piR-hsa-23210 associated diseases and relevant evidences.

Rank	disease name	Evidence
1	Cardiovascular diseases (CDC, CF, CCS) cardicregeneration	PMID: 28289238
2	Renal Cell Carcinoma	PMID: 25998508
3	Alzheimer Disease	PMID: 28127595
4	Male Infertility	PMID: 24855106
5	Gastric Cancer	PMID: 25779424

Note: the evidences can be found at https://pubmed.ncbi.nlm.nih.gov/.

**Table 7:** The top five piR-hsa-15023 associated diseases and relevant evidences.

Rank	disease name	Evidence
1	Cardiovascular diseases (CDC, CF, CCS) cardicregeneration	PMID: 28289238
2	Renal Cell Carcinoma	PMID: 26071182
3	Alzheimer Disease	PMID: 28127595
4	Rheumatoid Arthritis	None
5	Gastric Cancer	PMID: 25779424

Note: the evidences can be found at https://pubmed.ncbi.nlm.nih.gov/.

## Conclusion

In this study, we treat the task of piRNA-disease associations as a search task based on Learning to Rank [32, 68], where piRNA and disease are regarded as query and document, respectively. The following conclusions can be drawn: (i) iPiDA-LTR can effectively handle with two types of application scenarios compared with the other state-of-the-art methods, especially for the identification of diseases associated with newly detected piRNAs, which is important for studying the pathogenesis of disease and the function of piRNAs; (ii) iPiDA-LTR incorporates component methods into Learning to Rank so as to improve the predictive performance; (iii) The corresponding web server of iPiDA-LTR is freely accessed at http://bliulab.net/iPiDA-LTR/. Although iPiDA-LTR effectively predicts piRNA-disease associations, it only integrates basic machine learning methods and collaborative filtering. In future studies, we will integrate the other state-of-the-art methods and features to improve piRNA-disease associations. The LTR-based framework discussed in this study is a general framework, which would have many other applications in bioinformatics, such as protein function prediction, remote homology detection, etc.
